# Elevated dopamine alters consummatory pattern generation and increases behavioral variability during learning

**DOI:** 10.3389/fnint.2015.00037

**Published:** 2015-05-15

**Authors:** Mark A. Rossi, Henry H. Yin

**Affiliations:** ^1^Department of Psychology and Neuroscience, Duke UniversityDurham, NC, USA; ^2^Department of Neurobiology, Duke UniversityDurham, NC, USA; ^3^Center for Cognitive Neuroscience, Duke UniversityDurham, NC, USA

**Keywords:** dopamine transporter gene, reward, consummatory behavior, mouse, hyperdopaminergia

## Abstract

The role of dopamine in controlling behavior remains poorly understood. In this study we examined licking behavior in an established hyperdopaminergic mouse model—dopamine transporter knockout (DAT KO) mice. DAT KO mice showed higher rates of licking, which is due to increased perseveration of licking in a bout. By contrast, they showed increased individual lick durations, and reduced inter-lick intervals. During extinction, both KO and control mice transiently increased variability in lick pattern generation while reducing licking rate, yet they showed very different behavioral patterns. Control mice gradually increased lick duration as well as variability. By contrast, DAT KO mice exhibited more immediate (within 10 licks) adjustments—an immediate increase in lick duration variability, as well as more rapid extinction. These results suggest that the level of dopamine can modulate the persistence and pattern generation of a highly stereotyped consummatory behavior like licking, as well as new learning in response to changes in environmental feedback. Increased dopamine in DAT KO mice not only increased perseveration of bouts and individual lick duration, but also increased the behavioral variability in response to the extinction contingency and the rate of extinction.

## Introduction

Dopamine, a major neurotransmitter that mainly modulates activity in the basal ganglia, has been implicated in a variety of motivated behaviors (Zhou and Palmiter, 1995; Berridge, 2007; Rossi et al., 2013a,b). Extensive dopaminergic projections target the striatum, the input nucleus of the basal ganglia, where dopamine is released and influences the responsiveness of striatal neurons to glutamatergic cortical inputs (Gerfen and Surmeier, 2011). Dopamine release in the striatum is thought to be critical for the generation of orofacial behavior, including licking and chewing (Fray et al., 1980; Redgrave et al., 1980; Arnt et al., 1987; Delfs and Kelley, 1990; Skitek et al., 1999).

Previous studies have suggested a critical role of dopamine in appetitive and consummatory behaviors for food and water (Szczypka et al., 2001; Salamone and Correa, 2012). For example, systemic administration of dopamine receptor agonists potentiates orofacial movements and induces aberrant licking and chewing (Olpe, 1978; Zarrindast et al., 1992). Oral stereotypy can be blocked by pretreatment with dopamine receptor antagonists (Delfs and Kelley, 1990). Moreover, in dopamine transporter (DAT) knockout (KO) mice, which show prolonged increases in dopamine signaling (Giros et al., 1996), the overall rate of licking for sucrose reward was greatly increased (Costa et al., 2007). Nevertheless, exactly how dopamine can modulate consummatory behaviors remains unclear.

In this study, we used DAT KO mice, an established animal model of hyperdopaminergia (Giros et al., 1996; Rodriguiz et al., 2004), to study the role of dopamine in modulating consummatory behavior. Consummatory licking is a relatively stereotyped behavior in rodents, with tongue protrusions occurring at a rate of 5–9 Hz (Marowitz and Halpern, 1973; Halpern, 1975; Murakami, 1977; Weijnen, 1998). Despite the stereotypical pattern, however, studies have shown that the temporal structure of licking can be modulated by a variety of factors, such as perceptual feedback, satiety, palatability, and so on (Cone, 1974; Cone et al., 1975; Mamedov and Bures, 1984). However, the neural mechanisms that contribute to the patterning of licking behavior are largely unknown.

We quantified the pattern of licking in DAT KO mice and their wild type (WT) littermates using a contact lickometer as they voluntarily licked sucrose solution. We found that, compared to WT mice, DAT KO mice showed higher rates of licking, which were due to increased perseveration of licking in a bout. By contrast, they showed increased individual lick durations, and reduced inter-lick intervals. In addition, they also showed more rapid onset of behavioral variability following the onset of extinction and more rapid extinction of licking behavior.

## Materials and Methods

### Subjects

All procedures were conducted in accordance with the National Institutes of Health guidelines for the care and use of animals and in accordance with the Duke University Institutional Animal Care and Use Committee guidelines. DAT KO (*n* = 11) and WT (*n* = 8) littermates (8–12 weeks old) were used for experiments as previously described (Giros et al., 1996). During testing, mice were maintained on 23 h water deprivation schedule. Access to water was restricted to 1 h per day. All experiments were conducted during the light phase of the animal’s light cycle.

### Measuring Licking Behavior

Sucrose (10% w/v) was available from a standard water bottle in one wall of an operant chamber (35 cm × 28 cm × 22 cm). The spout was recessed ∼3 mm within a plastic tube to prevent the mice from contacting the lick sensor with their paws. Licks were recorded using a contact lickometer (Slotnick, 2009). When mice touched the metal spout with their tongues, a circuit was completed, which triggered a voltage change lasting the duration of the contact. Voltage was sampled at 2000 Hz using the Cerebus acquisition system (Blackrock Microsystems).

Testing took place in daily 30-min sessions. Thirsty mice were first acclimated to the testing chamber for 30 min and allowed to freely consume sucrose solution. The following day, mice were tested in the same chamber, and licks were recorded (sucrose condition). The next day, the extinction test was conducted in which the same set up was used, except the spout was empty. On the following day, reinstatement, sucrose solution was once again present in the spout. On the final day, the spout contained water. One KO mouse was omitted from the water test.

Locomotion tests were conducted after completion of the licking tests with a subset of the same mice used in the previous licking experiments (*n* = 8 WT; *n* = 9 KO). Mice were placed in a circular chamber for 3 min and video was taken from directly above. Position was tracked frame-by-frame oﬄine using custom software (Matlab) as described previously (Rossi et al., 2013a).

### Data Analysis

From the analog voltage signal, we generated timestamps corresponding to the onset and termination of each lick (0.5 ms resolution). From this we calculated the duration (time from onset to termination), duty cycle (the ratio of the duration of the contact with the lickometer to the period for each lick cycle), and inter-lick interval (ILI; time from lick offset to the next lick onset) as well as the number and rate of licks. Bout analysis was used to determine the properties of bouts of licking. Based on previous analysis of rodent licking (Spector et al., 1998), we defined the start of a bout as three or more licks occurring at >3 Hz and being preceded by at least 1 s in which no licks were recorded. The end of the bout was defined as the last lick that was followed by at least 1 s in which no licks were recorded. Power spectral density analysis was used to assess the component frequencies of the lick trains (Neuroexplorer, Nex Technologies). The lick rate was calculated as the inverse of the median ILI of all licks occurring within a bout.

For licking parameters that are not normally distributed (duration, ILI, duty cycle; Pearson omnibus normality test, *p* < 0.001), we used the median values to compare between groups. Median values were compared with Mann–Whitney test. To compare the number of licks per bout between groups, Welch’s *t*-test was used to correct for unequal variance.

## Results

### DAT KO Mice are Hyperactive and Show Altered Lick Patterning Relative to WT Controls When Freely Licking for Sucrose Solution

**FIGURE 1:**
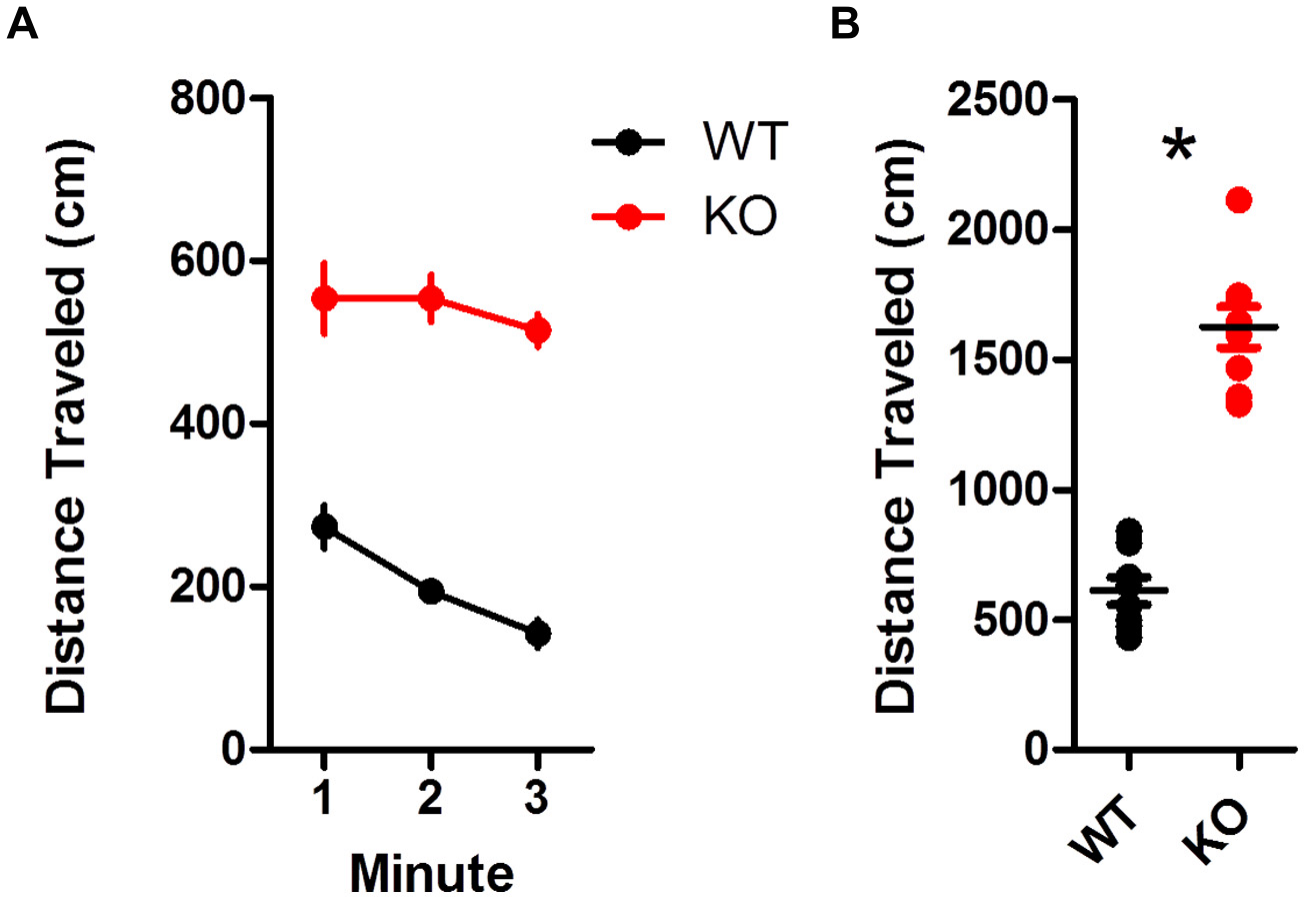
**Hyperactivity in dopamine transporter knockout (DAT KO) mice. (A)** Locomotion was tracked during 3 min in an open field chamber. KO mice showed no decline in locomotion during the test, while wild type (WT) mice did (left). **(B)** Overall, KO mice covered more distance than WT (^∗^*p* < 0.0001). Points represent data from individual mice. Lines represent mean and SEM.

Dopamine transporter knockout mice were hyperactive compared to WT mice during an open field test (**Figures 1A,B**). Overall, KO mice traveled much farther than WT littermates [**Figure 1B**; *t*_(15)_ = 10.33, *p* < 0.0001]. This is in agreement with previous studies showing hyperlocomotion in hyperdopaminergic mice (Giros et al., 1996).

**FIGURE 2:**
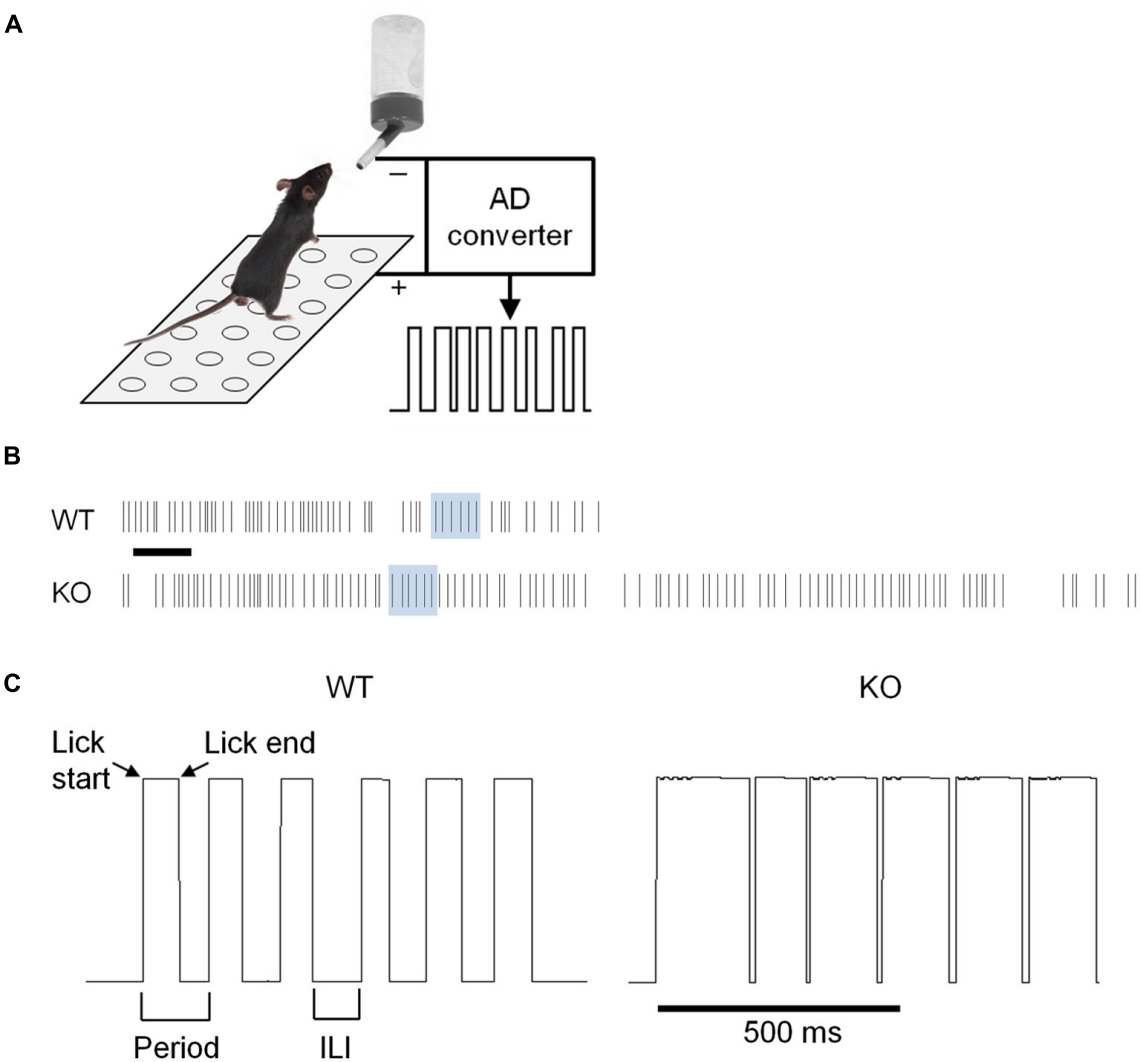
**Recording of voluntary licking in DAT KO mice. (A)** Schematic of apparatus. Mice stood on a metal ground plate and licked a spout that converted licks to a voltage signal. **(B)** Example traces from lickometer showing a sample bout of licking. Lick timestamps are shown as lines. Scale bar is 1 s. **(C)** Traces corresponding to the shaded regions from panel b showing individual licks as upward deflections for WT and KO mice. Note that the lick pattern is different in DAT KO mice.

**FIGURE 3:**
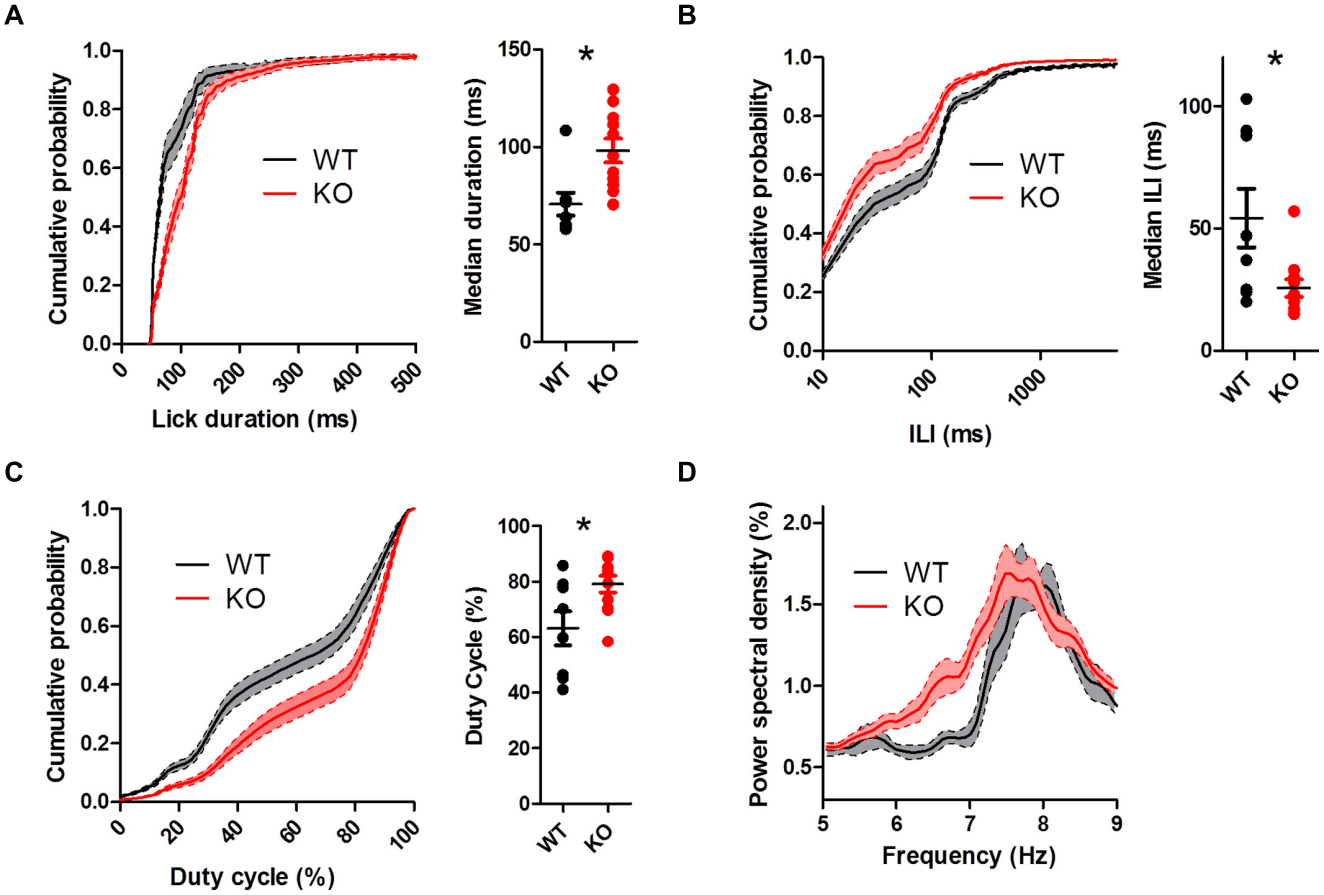
**Dopamine transporter knockout mice show altered lick patterning when licking sucrose. (A)** Distribution of lick durations. KO mice show longer durations than WT. **(B)** Distribution of inter-lick intervals (ILI). KO mice show shorter ILIs. **(C)** Distribution of duty cycles. KO mice show higher duty cycles than WT mice. **(D)** KO mice show elevated power spectral density at lower frequencies. ^∗^*p* < 0.05. Points represent data from individual mice. Lines represent mean and SEM.

To assess whether chronically elevated dopamine alters consummatory motor output, we designed a contact lickometer that detected individual licks (**Figure 2A**; Slotnick, 2009). We found that DAT KO mice had altered lick patterning compared to WT mice when voluntarily licking for sucrose solution (**Figure 2B,C**). Overall, the lick cycle of DAT KO mice (within bout rate: 7.66 ± 0.13 Hz) appeared slower relative to WT mice (within bout rate: 8.28 ± 0.20 Hz). DAT KO mice showed longer lick durations than WT mice (**Figure 3A**; Mann–Whitney *U* = 10.00, *p* = 0.0057) and shorter ILIs (**Figure 3B**; *U* = 17.00, *p* = 0.028). The duty cycle (the ratio of the duration of the contact with the lickometer to the period for each lick cycle) was increased in DAT KO mice relative to WT mice (**Figure 3C**; *U* = 19.00, *p* = 0.043).

To further examine the pattern of licking, we used power spectral density analysis to extract the component frequencies of bouts of licking (**Figure 3D**). DAT KO mice showed higher power at lower lick frequencies compared to WT mice [two-way ANOVA (Genotype × Frequency): main effect of Genotype, *F*_(1,850)_ = 19.82, *p* = 0.0004; main effect of Frequency, *F*_(50,850)_ = 25.68, *p* < 0.000; interaction between Genotype and Frequency, *F*_(50,850)_ = 1.65, *p* = 0.0036].

**FIGURE 4:**
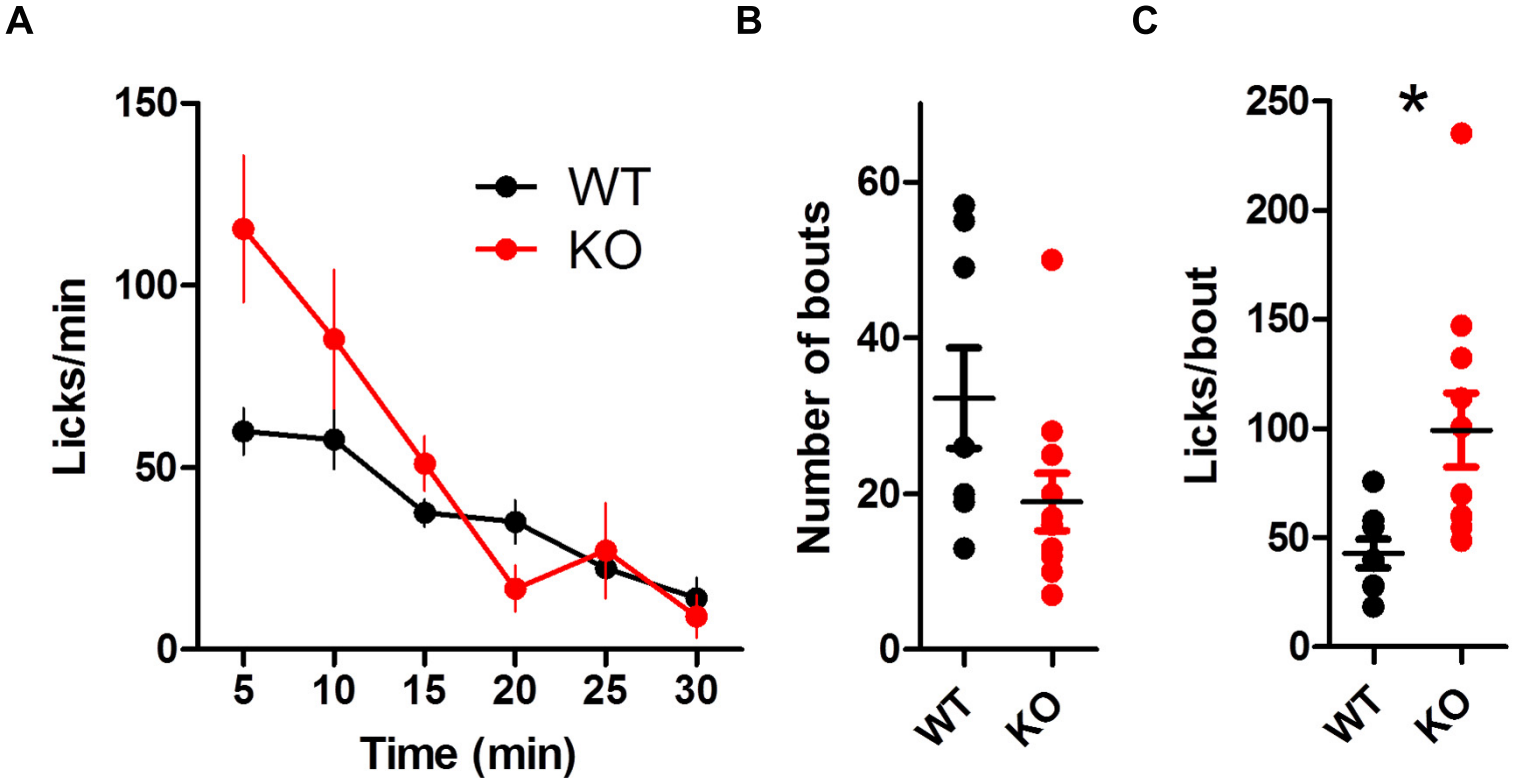
**Dopamine transporter knockout mice show increased rate of licking and number of licks in a bout. **(A)** Rate of licking throughout the session. (B)** Number of bouts. **(C)** DAT KO mice lick more in each bout. ^∗^*p* < 0.05. Points represent data from individual mice. Lines represent mean and SEM.

The overall lick rate was increased for KO mice [**Figure 4A**; two-way ANOVA (Genotype × Time): no main effect of Genotype, *F*_(1,85)_ = 3.32, *p* = 0.086; main effect of Time, *F*_(5,85)_ = 13.37, *p* < 0.0001; interaction between Genotype and Time, *F*_(5,85)_ = 2.57, *p* = 0.032]. The interaction was driven by more licks for KO mice during the first 5 min of the session (Bonferroni post test, *p* < 0.01). DAT KO mice took marginally fewer bouts than WT mice [**Table 1**; **Figure 4B**; *t*_(17)_ = 1.92, *p* = 0.07] but had more licks per bout than WT mice [**Figure 4C**; unpaired *t*-test with Welch’s correction for unequal variance: *t*_(12)_ = 3.10, *p* = 0.0092].

**Table 1:** Mean ± SEM number of bouts at different pause criteria.

	0.3 s	1 s	3 s	10 s
**Sourose**				
WT	63.50 ± 11.43	32.25 ± 6.44	22.37 ± 4.21^∗^	16.37 ± 2.82^∗^
KO	74.90 ± 12.69	18.90 ± 3.67	11.09 ± 1.42	8.91 ± 1.01
**Extinction**				
WT	78.75 ± 14.19	53.87 ± 8.97^∗^	37.25 ± 5.51^∗^	23.38 ± 2.43^∗^
KO	43.09 ± 13.33	19.09 ± 5.47	12.72 ± 2.39	10.82 ± 2.07
**Reinstatement**				
WT	48.50 ± 4.65	31.00 ± 3.28	22.75 ± 2.90	19.00 ± 2.30^∗^
KO	72.54 ± 14.87	25.81 ± 6.78	15.00 ± 4.30	10.91 ± 2.38
**Water**				
WT	60.12 ± 11.63	36.62 ± 5.57^∗^	26.37 ± 3.35^∗^	18.75 ± 2.01^∗^
KO	63.80 ± 11.92	20.70 ± 3.81	13.70 ± 2.89	10.70 ± 2.13

Criteria are based on (Spector et al., 1998). *p < 0.05, t-test between KO and WT.

### In Extinction, in Which no Solution is Present, DAT KO Mice no Longer Show Altered Lick Patterning Relative to WT Controls

**FIGURE 5:**
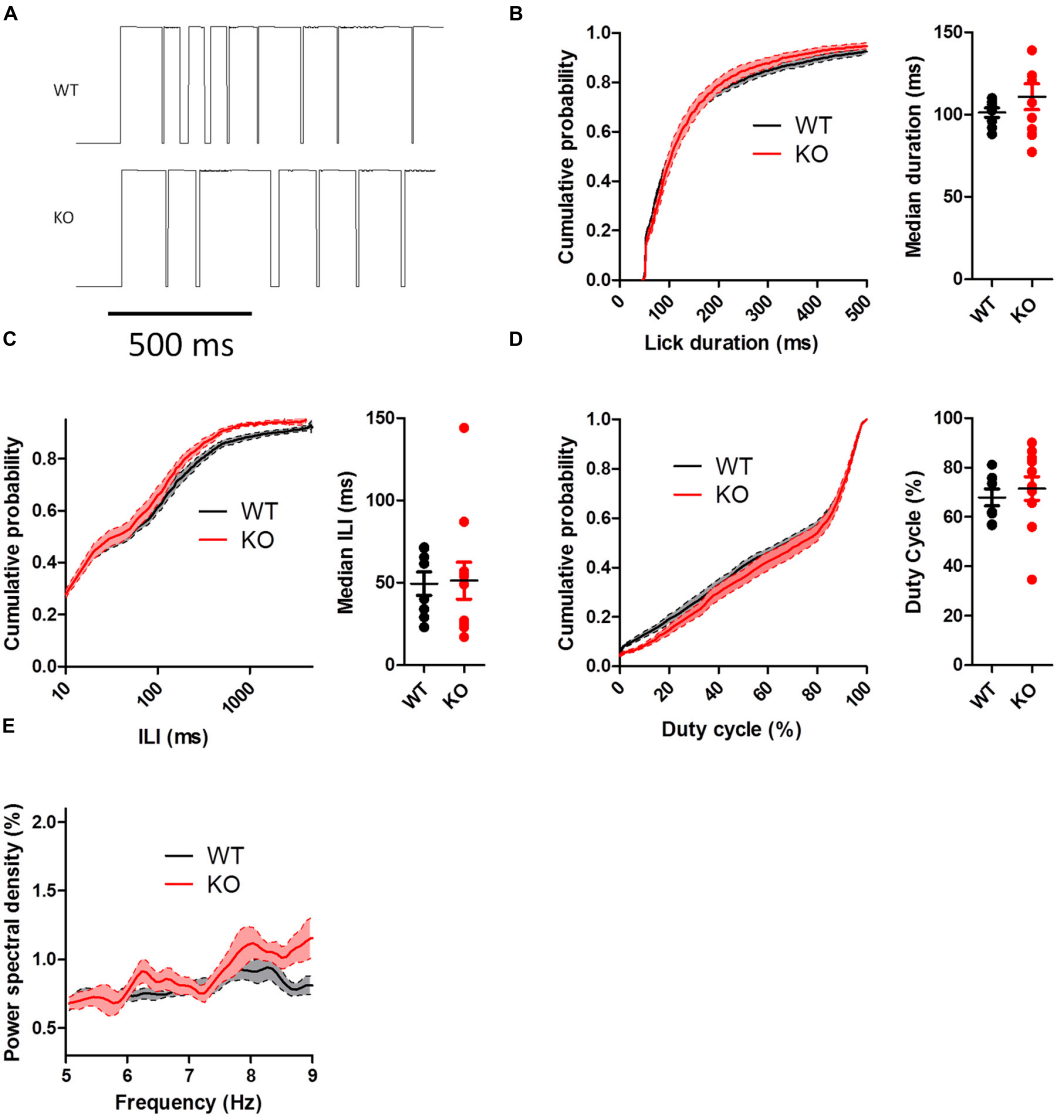
**Lick patterning during extinction. (A)** Representative lickometer traces for WT (top) and KO (bottom) mice. When the data from the entire extinction session were compared, neither lick duration **(B)**, ILI **(C)**, nor duty cycle **(D)** were different between WT and KO mice when licking in extinction. **(E)** Power spectral density of licking. Points represent data from individual mice. Lines represent mean and SEM.

**FIGURE 6:**
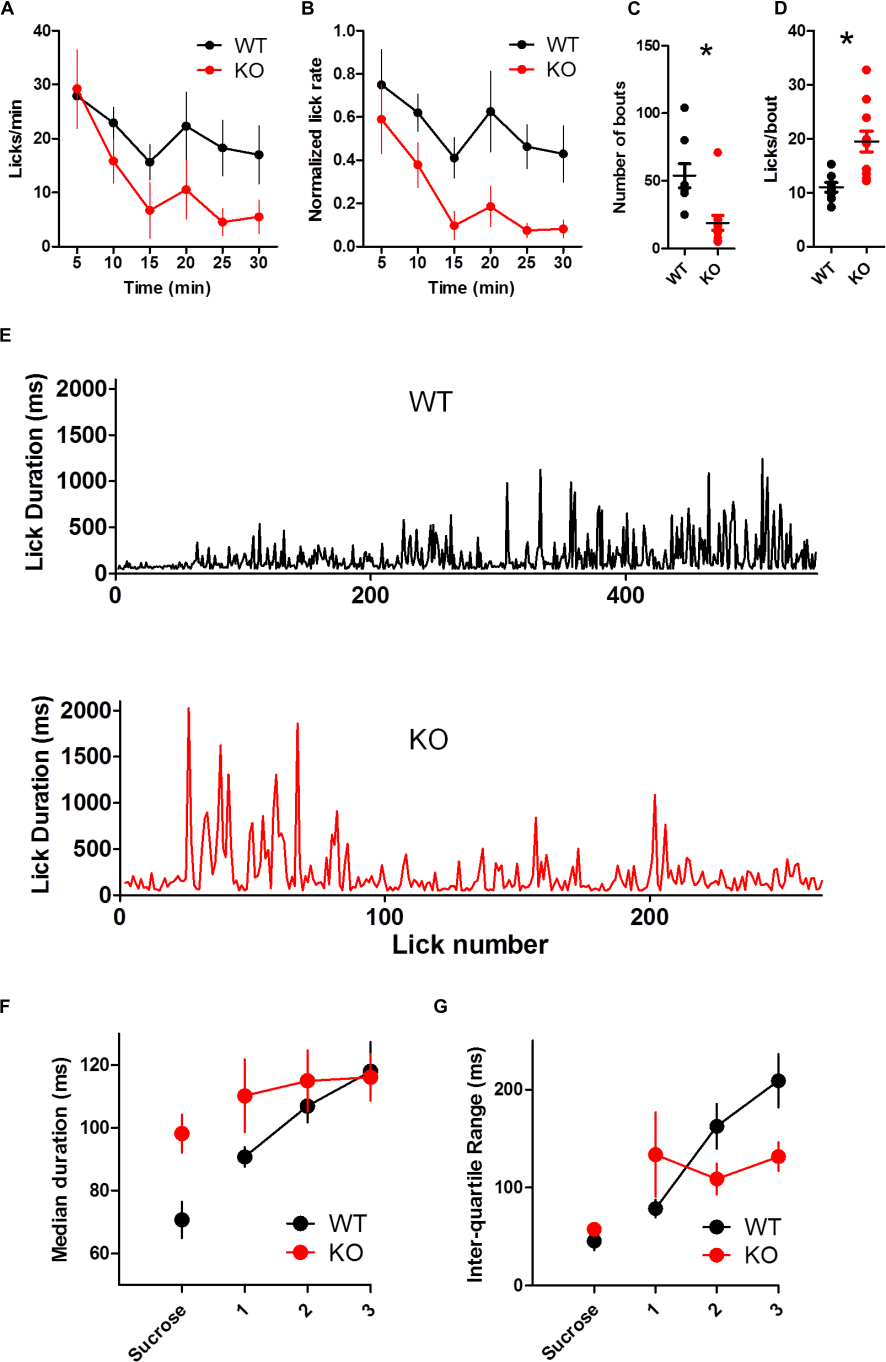
**Dopamine transporter knockout mice are more sensitive than WT mice to extinction. (A)** Rate of licking throughout the 30-min extinction session. **(B)** Rate of licking throughout the session normalized by the average rate of licking for sucrose. KO mice reduce their lick rate more quickly than WT mice. KO mice show fewer bouts of licking **(C)** and more licks per bout **(D)**. **(E)** Representative examples of lick duration throughout the session from WT (top) and KO (bottom) mice. **(F)** Median duration of licks. Sucrose indicates data from the last rewarded session. **(G)** Inter quartile range of lick duration. ^∗^*p* < 0.05. Points represent data from individual mice. Lines represent mean and SEM.

To test whether the differences in the patterning of DAT KO licking were influenced by feedback related to the presence of sucrose in the spout, we conducted licking tests in extinction, when no solution was present. When mice licked the dry spout, the rate of licking was slower than when they licked for sucrose (WT: 6.47 ± 0.21 Hz; KO: 6.32 ± 0.23 Hz). In addition, the observed differences in the pattern of licking disappeared (**Figures 5** and **6**). During extinction, DAT KO mice no longer had increased lick durations (**Figure 5B**; *U* = 38.50, *p* = 0.68), shorter ILIs (**Figure 5C**; *U* = 35.5, *p* = 0.51), or higher duty cycles (**Figure 5D**; *U* = 33.00, *p* = 0.39). Neither group showed enhanced power within the 7–9 Hz range (**Figure 5E**).

### DAT KO Mice are More Sensitive Than WT Controls to Extinction

Knockout mice were more sensitive to extinction than WT mice (**Figure 6**). Both groups reduced the rate of licking during extinction [**Figure 6A**; two-way ANOVA (Genotype × Time): no main effect of Genotype, *p* > 0.05; main effect of Time, *F*_(5,85)_ = 7.12, *p* < 0.0001; no Interaction, *p* > 0.05]. When the lick rate was normalized by the average rate of licking during the preceding sucrose session, KO mice extinguished licking more quickly than WT mice [**Figure 6B**; two-way ANOVA (Genotype × Time): main effect of Genotype, *F*_(1,85)_ = 9.23, *p* = 0.0074; main effect of Time, *F*_(5,85)_ = 6.79, *p* < 0.0001; no interaction between Genotype and Time, *F*_(5,85)_ = 0.61, *p* = 0.69].

Dopamine transporter knockout mice took fewer bouts than WT mice [**Figure 6C**; *t*_(17)_ = 3.50, *p* = 0.0028]. The number of licks per bout was greatly reduced for both groups compared to the sucrose condition, yet KO mice still tended to lick more per bout [**Figure 6D**; *t*-test with Welch’s correction for unequal variance: *t*_(13)_ = 3.97, *p* = 0.0016].

To understand the time course of behavioral adaptation to altered feedback during extinction, we analyzed how lick duration changed over time (**Figure 6E–G**). DAT KO mice adapted more quickly to the dry spout than WT mice. Because all mice licked different amounts, we divided the licks of each mouse into three equally sized bins to compare lick duration and variability throughout the extinction session. The duration of licks was stable throughout the session, and there was no group difference [**Figure 6F**; two wan ANOVA (Genotype × Time): no main effect of Genotype, *F*_(1,34)_ = 0.75, *p* > 0.05; no main effect of Time, *F*_(2,34)_ = 2.98, *p* = 0.06; no interaction, *F*_(2,34)_ = 1.21, *p* > 0.05]. In response to the dry spout, mice increased the variability of lick durations. DAT KO mice, however, did so more quickly than WT mice as measured by the inter quartile range (**Figure 6G**; no main effect of Genotype, *F*_(1,34)_ = 1.06, *p* > 0.05; main effect of Time, *F*_(2,34)_ = 3.53, *p* = 0.04; interaction between Genotype and Time, *F*_(2,34)_ = 4.27, *p* = 0.02). DAT KO mice had reduced variability late in the extinction test relative to WT mice (*p* < 0.05).

### Altered Lick Patterning in KO Mice is Restored When Sucrose is Reinstated

**FIGURE 7:**
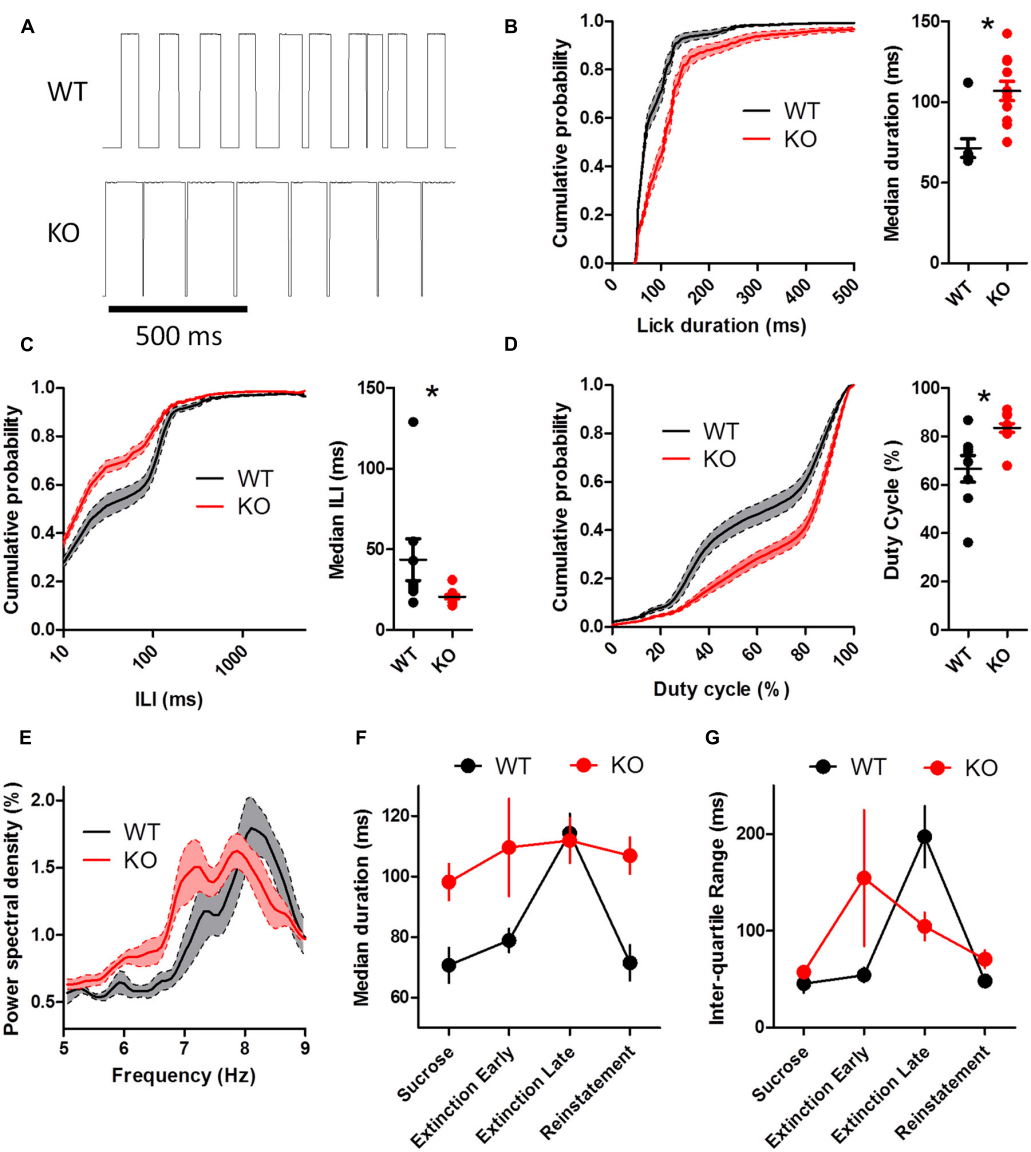
**Altered lick patterning in DAT KO mice during reinstatement. (A)** Representative lickometer traces for WT (top) and KO (bottom) mice. Lick duration **(B)**, ILI **(C)**, and duty cycle **(D)** are all different between WT and KO mice when licking for sucrose solution is reinstated. **(E)** Power spectral density of licking returns to pre-extinction levels. **(F,G)** KO mice adapt licking behavior to altered feedback during extinction more quickly than WT mice. The first 50 licks during extinction was considered ‘early’ and the last 50 licks was considered ‘late.’ Duration of licks **(F)** and inter-quartile range of lick durations **(G)** returns to pre-extinction levels during reinstatement. ^∗^*p* < 0.05. Points represent data from individual mice. Lines represent mean and SEM.

Following extinction, licking for sucrose solution was reinstated (**Figure 7**). Licking during reinstatement was highly similar to pre-extinction licking (**Figure 7A**; WT: 8.36 ± 0.21 Hz; KO: 7.60 ± 0.20 Hz). DAT KO mice had longer lick durations (**Figure 7B**; *U* = 7.00, *p* = 0.0026), shorter ILIs (**Figure 7C**; *U* = 12.50, *p* = 0.01), and higher duty cycles (**Figure 7D**; *U* = 12.00, *p* = 0.0093) than WT mice. Enhanced power was also observed in the 7–9 Hz frequency range (**Figure 7E**).

We compared the effects of extinction early in the session (first 50 licks) with the last 50 licks (**Figures 7F,G**>). Lick duration was altered by extinction and returned to pre-extinction levels during reinstatement (**Figure 7F**; main effect of Genotype, *F*_(1,51)_ = 9.03, *p* = 0.008; main effect of Time, *F*_(3,51)_ = 4.74, *p* = 0.005; no interaction between Genotype and Time, *F*_(3,51)_ = 2.20, *p* = 0.10). The inter quartile range of the lick durations was also altered by extinction and returned to pre-extinction levels during reinstatement, but WTs adapted more slowly (**Figure 7G**; no main effect of Genotype, *F*_(1,51)_ = 0.19, *p* = 0.67; main effect of Time, *F*_(3,51)_ = 4.41, *p* = 0.008; interaction between Genotype and Time, *F*_(3,51)_ = 3.29, *p* = 0.03). KO mice had reduced lick variability during the late phase of extinction relative to WT mice (*p* < 0.05).

**FIGURE 8:**
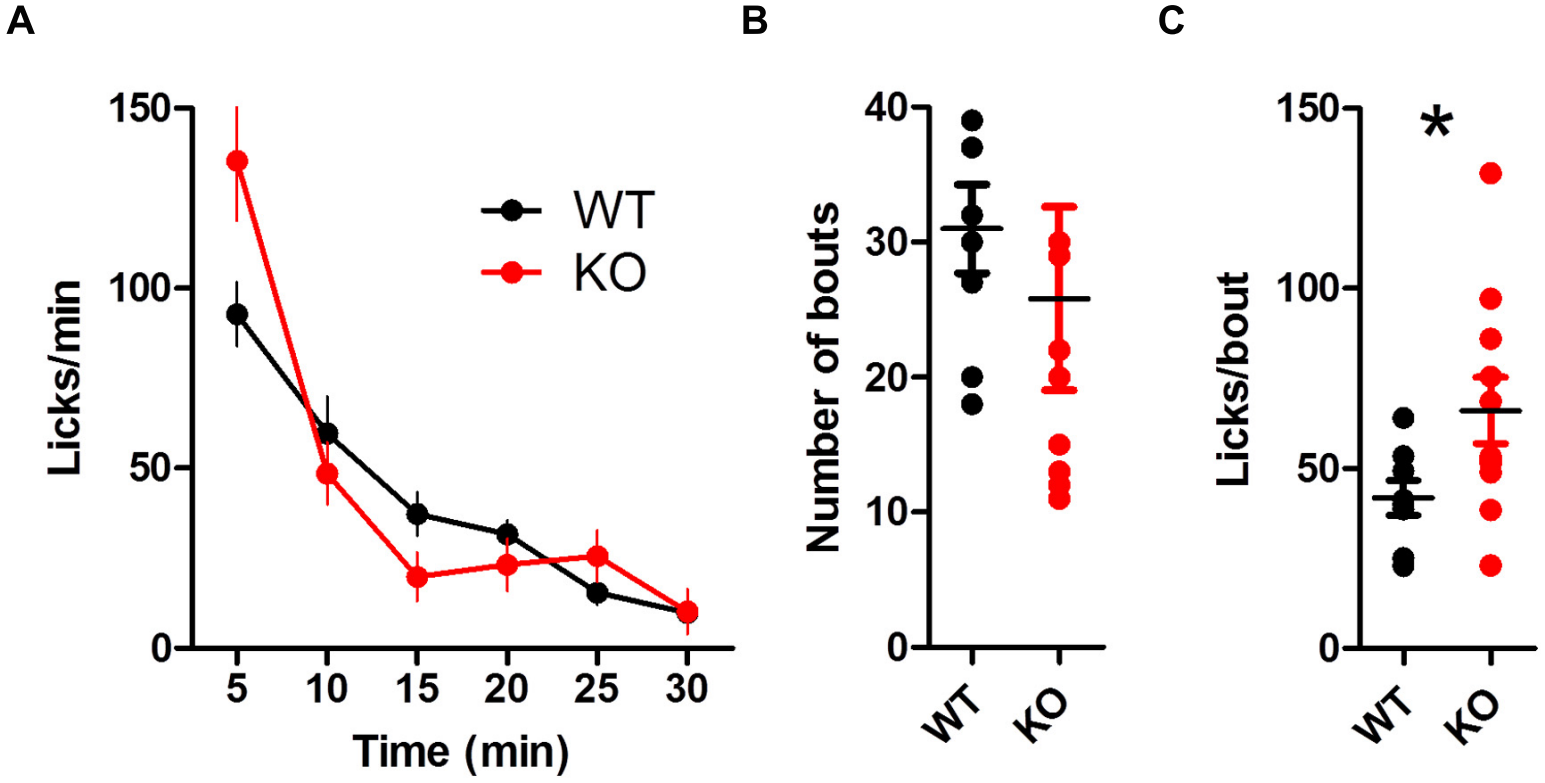
**Summary of licking during reinstatement. (A)** Rate of licking throughout the session is similar between groups. **(B)** The number of bouts is similar between groups. **(C)** DAT KO mice lick more in each bout, ^∗^*p* < 0.05. Points represent data from individual mice. Lines represent mean and SEM.

The global rate of licking was similar between groups [**Figure 8A**; two-way ANOVA (Genotype × Time): no main effect of Genotype, *F*_(1,85)_ = 0.85, *p* = 0.37; main effect of Time, *F*_(5,85)_ = 36.56, *p* < 0.0001; no interaction between Genotype and Time, *F*_(5,85)_ = 1.23, *p* = 0.30]. Both groups took a similar number of bouts (**Figure 8B**; *t*_(17)_ = 0.55, *p* > 0.05). DAT KO mice had more licks per bout during reinstatement (**Figure 8C**; unpaired *t*-test with Welch’s correction for unequal variance: *t*_(14)_ = 2.33, *p* = 0.035).

### Lick Patterning is Similar Between KO and WT Mice When Licking Water

**FIGURE 9:**
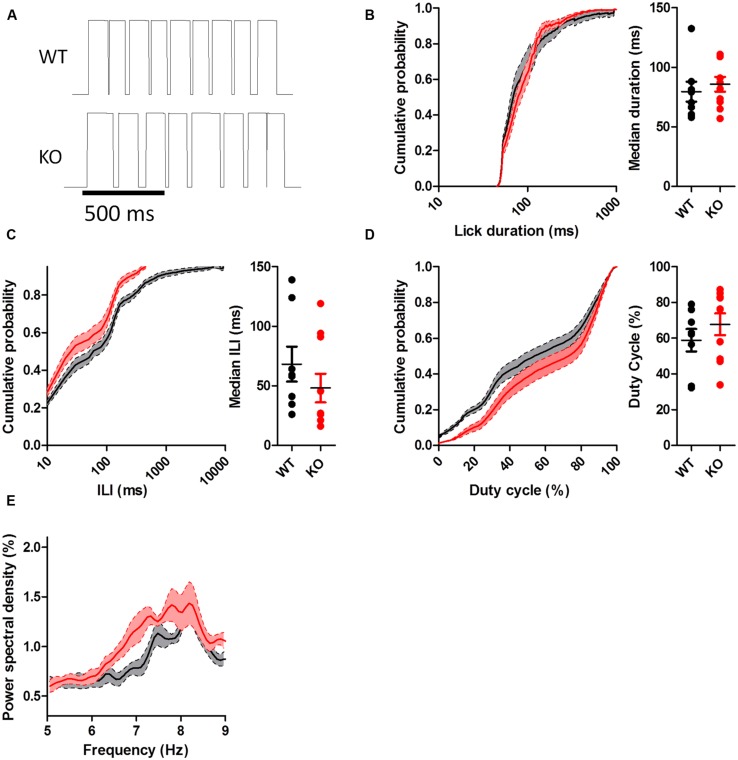
**There was no significant difference in lick patterning when licking water. (A)** Representative lickometer traces for WT (top) and KO (bottom) mice. Lick duration **(B)**, ILI **(C)**, and duty cycle **(D)** are similar between WT and KO mice when licking for water (*p* > 0.05). **(E)** Power spectral density of licking. Points represent data from individual mice. Lines represent mean and SEM.

To test whether the observed differences in the temporal structure of licking were due to the high incentive value associated with sucrose solution, we recorded licking while mice consumed water. We found that licking was much more similar between DAT KO and WT mice than when they were licking for sucrose (**Figure 9A**; WT: 7.67 ± 0.28 Hz; KO: 7.96 ± 0.13 Hz). There was no statistically significant difference between KO and WT mice in lick duration (**Figure 9B**; *U* = 30.00, *p* = 0.41), ILIs (**Figure 9C**; *U* = 22.50, *p* = 0.13), or duty cycle (**Figure 9D**; *U* = 25.00, *p* = 0.20). Both groups showed enhanced power within the 7–9 Hz range (**Figure 9E**).

**FIGURE 10:**
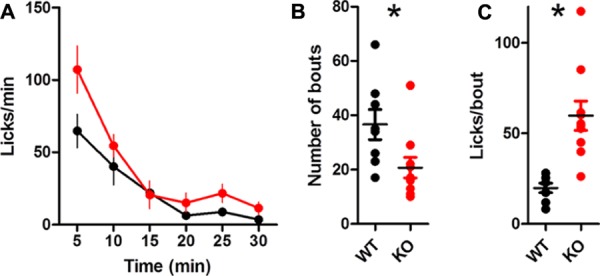
**Summary of licking for water. (A)** Rate of licking throughout the session. **(B)** DAT KO mice have fewer bouts of licking. **(C)** KO mice lick more in each bout, ^∗^*p* < 0.05. Points represent data from individual mice. Lines represent mean and SEM.

When licking water, the global lick rate was similar between groups [**Figure 10A**; no main effect of Genotype, *F*_(1,80)_ = 3.50, *p* = 0.08; main effect of Time, *F*_(5,80)_ = 31.46, *p* < 0.0001; no interaction between Genotype and Time, *F*_(5,80)_ = 1.90, *p* = 0.10]. DAT KO mice took fewer bouts [**Figure 10B**; *t*_(16)_ = 2.43, *p* = 0.027]. DAT KO mice had more licks per bout than WT [**Figure 10C**; unpaired *t*-test with Welch’s correction for unequal variance: *t*_(10)_ = 4.71, *p* = 0.0008].

## Discussion

The DAT KO is a well-established mouse model of hyperdopaminergia (Giros et al., 1996; Spielewoy et al., 2000). In agreement with previous work, we found general hyperactivity in these mice. Our novel finding is a pronounced change in the pattern and timing of licking. DAT KOs showed increased individual lick duration, reduced ILI, and more licks per bout. In addition, when the feedback was altered during extinction, we found a dramatic difference in the consequent change in licking behavior: whereas controls showed a gradual increase in behavioral variability over time, the DAT KOs were much more sensitive to the change in the feedback function, showing an initial, transient burst of variability following the detection of the extinction contingency (**Figure 6G**). This more rapid generation of behavioral variability is also coupled with faster extinction (**Figure 6B**).

### Generation of Licking Pattern

In recent years, licking behavior has increasingly become a focus of neuroscience research because it is highly reliable and stereotyped, permitting convenient characterization of the temporal structure of behavior (Gutierrez et al., 2010; Johnson et al., 2010; Komiyama et al., 2010; Ostlund et al., 2013; Guo et al., 2014).

Although the pattern generator underlying licking is well characterized, the top–down influences, including the role of the basal ganglia, remain largely unknown. Some cortical regions are known to show lick related activity (Gutierrez et al., 2006; Komiyama et al., 2010). Neurons from these regions may drive licking via striatal projections. The neurons within the lateral striatum exhibit oscillatory activity that corresponds to licking behavior (Mittler et al., 1994). Injection of amphetamine in the ventrolateral striatum induces oral stereotypy and voracious licking (Kelley et al., 1988), and unilateral depletion of nigrostriatal dopamine in rats results in impaired licking in which the lick rate slows and tongue force is reduced (Skitek et al., 1999).

Output from the basal ganglia is thought to regulate orofacial movements via projections to the motor regions of the superior colliculus (Taha et al., 1982; Gunne et al., 1988). The superior colliculus, in turn, sends projections to rhythmically active motor and premotor neurons in the reticular formation that control the tongue and jaws (Morimoto et al., 1966; Wiesenfeld et al., 1977; Sparks and Hartwich-Young, 1989; Brozek et al., 1996; Travers et al., 1997).

Licking is often considered a highly stereotyped behavior controlled by a central pattern generator. However, as shown in the present study, the pattern is certainly influenced by feedback from the liquid during licking. DAT KO mice showed higher overall rates of licking for sucrose. This result is not surprising given the known hyperactivity in these mice (Costa et al., 2007; Perona et al., 2008), but we showed for the first time a significant change in the patterning of licking. These changes are characterized by a dramatic increase in the persistence of a bout of licking (more licks per bout), as well as an increase in the individual lick contact duration. Their high rate of licking is primarily due to an increase in the number of licks per bout, i.e., more persistent licking once the bout is initiated. On the other hand, during each bout, their licking is actually slower, characterized by longer duration of contact with the spout and shorter ILIs. This observation can be explained by the hypothesis that DAT KO mice attempt to maximize sucrose intake. In accord with this interpretation, the overall proportion of contact time during a bout (duty cycle) is increased in the DAT KO group.

### Extinction

Here we showed that in control mice, the lick duration increased in extinction, when the sucrose reward was no longer delivered. WT mice gradually increased variability of lick duration during extinction, as shown in **Figure 6G**. Extinction, as a procedure, represents a drastic change in the feedback function, and has long been shown to result in new learning. As shown by the rate of licking, all mice reduced their overall rate of licking over time, but the DAT KO mice showed a more rapid reduction (**Figure 6B**). Moreover, they immediately increased behavioral variability, whereas the controls showed a more gradual change.

An increase in behavioral variability is present in initial instrumental learning (Derusso et al., 2010; Costa, 2011; Yin, 2014a). But such variability could be a general feature of learning, including the behavioral adaptations following exposure to the extinction contingency. The mice learned to stop licking, but initially they exhibited “exploration” by varying the pattern and timing of licking. Despite the highly stereotyped licking pattern, both the ILI and the lick duration could be varied, and all mice increased such behavioral variability following the onset of extinction.

### The Function of Dopamine

Our results are in accord with previous work showing a critical role of dopamine in the performance of orofacial movements. For example, studies have shown reduced lick frequency in rats with unilateral striatal dopamine depletion (Skitek et al., 1999) and increased frequency of orofacial movements following systemic or intrastriatal administration of dopamine agonists (Fray et al., 1980; Redgrave et al., 1980; Arnt et al., 1987; Delfs and Kelley, 1990). Yet our results suggest that increasing dopamine transmission produces two distinct types of effects—one related to performance and the other related to learning.

The net effect of dopamine is to modulate the gain of the reward seeking system, altering performance to maximize the input by prolonging the contact duration (duty cycle). This suggests that dopamine could be operating at a hierarchically higher level that has access to net sucrose intake. This level, presumably corresponding to the basal ganglia circuits, can simultaneously modulate all three effects (ILI, contact duration, and bout persistence) in the right direction to maximize sucrose intake. As a result, the overall sucrose yield per bout of licking is increased. This interpretation is also supported by the finding that the difference between KO mice and controls is much reduced when they are licking for water, which has lower incentive value (**Figure 9**).

It has recently been proposed that the basal ganglia networks are closed loop controllers that regulate transition or rate of change in different perceptual variables (Yin, 2014a,b,c; Barter et al., 2015; Rossi et al., 2015). Dopaminergic projections to the striatum can adjust the gain of such a system. The limbic and associative cortico-basal ganglia networks can be especially important for the control of reward rates using diverse behavioral outputs (Yin et al., 2008; Yin, 2014a).

The second effect is related to learning, in this case to behavioral adaptation during extinction or non-reinforcement. As has long been established, extinction can result in new types of learning (Bouton, 2004). Here dopamine appears to modulate the level of behavioral variability in licking pattern generation (Costa, 2011). This effect is in agreement with work on dopaminergic modulation of song variability in song birds (Leblois et al., 2010; Leblois and Perkel, 2012). The prolonged dopamine signaling in DAT KO mice may therefore increase variability depending on the behavioral context, whether it is modulation of song production based on social context or the modulation of licking variability following the onset of extinction. Because the generation of such behavioral variability is critical during the exploratory phase of learning, dopamine can also play a key role in learning.

## Conflict of Interest Statement

The authors declare that the research was conducted in the absence of any commercial or financial relationships that could be construed as a potential conflict of interest.
